# Sodium-Glucose Cotransporter-2 Inhibitor (SGLT2i) as a Primary Preventative Agent in the Healthy Individual: A Need of a Future Randomised Clinical Trial?

**DOI:** 10.3389/fmed.2021.712671

**Published:** 2021-08-23

**Authors:** Dan Xu, Owain Chandler, Cleo Wee, Chau Ho, Jacquita S. Affandi, Daya Yang, Xinxue Liao, Wei Chen, Yanbing Li, Christopher Reid, Haipeng Xiao

**Affiliations:** ^1^Faculty of Health Sciences, CCRE, Curtin School of Population Health, Curtin University, Perth, WA, Australia; ^2^Faculty of Health Sciences, Curtin Medical School, Curtin University, Perth, WA, Australia; ^3^Department of Medical Education, First Affiliated Hospital, Sun Yat-sen University, Guangzhou, China; ^4^Department of Cardiology, First Affiliated Hospital, Sun Yat-sen University, Guangzhou, China; ^5^Department of Renal Medicine, First Affiliated Hospital, Sun Yat-sen University, Guangzhou, China; ^6^Department of Endocrinology, First Affiliated Hospital, Sun Yat-sen University, Guangzhou, China

**Keywords:** SGLT2i inhibitor, primary prevention, cardioprotection, renoprotection, chronic diseases prevention

## Abstract

Sodium-glucose cotransporter-2 inhibitors (SGLT2i) are a relatively novel class of drug for treating type 2 diabetes mellitus (T2DM) that inhibits glucose reabsorption in the renal proximal tubule to promote glycosuria and reduce blood glucose levels. SGLT2i has been clinically indicated for treating T2DM, with numerous recent publications focussing on both primary and secondary prevention of cardiovascular and renal events in Type 2 diabetic patients. The most recent clinical trials showed that SGLT2i have moderately significant beneficial effects on atherosclerotic major adverse cardiovascular events (MACE) in patients with histories of atherosclerotic cardiovascular disease. In this review and analysis, SGLT2i have however demonstrated clinically significant benefits in reducing hospitalisation for heart failure and worsening of chronic kidney disease (CKD) irrespective of pre-existing atherosclerotic cardiovascular disease or previous heart failure history. A meta-analysis suggests that all SGLT2 inhibitors demonstrated the therapeutic benefit on all-cause and cardiovascular mortality, as shown in EMPAREG OUTCOME study with a significant decrease in myocardial infarction, without increased stroke risk. All the above clinical trial recruited type 2 diabetic patients. This article aims to postulate and review the possible primary prevention role of SGLT2i in healthy individuals by reviewing the current literature and provide a prospective overview. The emphasis will include primary prevention of Type 2 Diabetes, Heart Failure, CKD, Hypertension, Obesity and Dyslipidaemia in healthy individuals, whom are defined as healthy, low or intermediate risks patients.

## Introduction

SGLT2i is a novel class of medication that decreases plasma glucose concentration. The pharmacological action shows that a healthy individual will approximately filter 160–180g glucose daily from the glomerulus with almost 100% reabsorption via the proximal convoluted tubule (PCT), hence leaving almost no urinary glucose. Diabetic patients' glomerular filtration of glucose increased significantly to 180–240g daily above renal glucose threshold, leading to glycosuria. SGLT2i bind to SGLT2 receptors with an insulin-independent mechanism of action, further increasing glycosuria and consequently lowering plasma glucose concentration (1), leading to cardiovascular and renal benefit demonstrated in systematic review and meta-analysis (1, 2). Benefits of SGLT2i mechanism of action include improvements in fasting as well as postprandial hyperglycaemia, decreased risk of hypoglycaemia, reduced weight gain, lowering blood pressure and decreased fluid retention (3). SGLT2i also helps to prevent the long-term complications of continually raised HBA1c levels (4). There is currently no global consensus guideline for the indication of SGLT2i use in different regions and countries. We reviewed guidelines and recommendations for SGLT2i use in countries with a large population of type 2 diabetes (Table 1) in this narrative review aiming to address whether a future RCT is needed to examine the potential primary preventive role of SGLT2i in healthy individual.

**Table 1:** Summary of SGLT2i guideline in different countries.

	**Australian guideline**	**The US guideline**	**China guideline**	**The UK and EU countries**
Approved SGLT2i use	Dapagliflozin Empagliflozin (Canagliflozin was delisted from the PBS due to pricing issue)	Dapagliflozin Empagliflozin Canagliflozin Ertugliflozin	Dapagliflozin Empagliflozin Canagliflozin Sotagliflozin (the first dual SGLT2/1 inhibitor)	Dapagliflozin Empagliflozin Canagliflozin
Recommendations	DM patients: SGLT2 as an add on therapy to metformin.			SGLT2 as an add on therapy to metformin in DM patients, and as a first line therapy in patients with DM+Established CVD.
	Patients with established CVD or two or more CVD risk factors	Patients with established CVD or high CVD risk or established CKD or HF	Consider SGLT2i in high CVD risk or HF patients.	
Concerns	Caution and review use with diuretics, caution with Dehydration, Genitourinary infections, Euglycemic diabetic ketoacidosis, Weight loss	Caution with severe renal impairment, Dehydration, Genitourinary infections, Euglycemic diabetic ketoacidosis	Not recommend SGLT2i for patients with severely impaired renal function, however dosage adjusting in those with impaired renal function.	Not recommend SGLT2i for patients with kidney disease.

## Methods

The authors performed an extensive literature review using the following Medical Subject Headings (MeSH) terms including SGLT2i, guideline, safety, cardiovascular diseases, chronic kidney diseases, heart failure, hypertension, dyslipidaemias, obesity, chronic diseases and primary prevention. The review has identified 2185 publications related to SGLT2 inhibitors' safety, guidelines in different countries and potential preventive role in cardiovascular diseases, chronic kidney diseases, heart failure, hypertension, dyslipidaemias, obesity and chronic diseases. After scanning the titles and the abstracts to remove duplication, we have included 88 publications based on SGLT2 inhibitors' relevancy on the role of primary prevention by using the Oxford Equator PRISMA checklist to satisfy an evidence-based narrative review.

## SGLT2i Use and Its Guideline

In Australia, the Therapeutic and Goods Administration (TGA), has currently approved two SGLT2i use in Australia: dapagliflozin and empagliflozin (5). Canagliflozin was delisted from the Pharmaceutical Benefit Scheme (PBS) in 2015 due to pricing issue (6). The use of SGLT2i is recommended to combine with metformin in treating T2DM patients, as well as considered use in diabetic patients with cardiovascular diseases or at least two cardiovascular risk factors (7). SGLT2i has the greatest therapeutic effect in those with preserved renal function, with declining efficacy as Glomerular Filtration Rate (GFR) decreases (3).

In the United States of America, the Food and Drug Administration (FDA) has authorised the use of four SGLT2 inhibitors including canagliflozin, dapagliflozin, empagliflozin and ertugliflozin in treating T2DM patients. Recent guidelines by American Diabetes Association state that patients with high-risk or pre-existing atherosclerotic cardiovascular disease, or pre-existing kidney disease, or heart failure, we recommended SGLT2i as part of the glucose-reducing regimen irrespective of HbA1c control (8).

In the United Kingdom and the European Union, SGLT2 inhibitors are approved as an add-on therapy for patients having high blood glucose levels despite being on metformin and insulin. They are, however, not recommended to be prescribed for patients with CKD due to decreased efficacy (9).

In the European continent, SGLT2i is indicated in the 2019 European Society of Cardiology guideline as first-line therapy for patients with T2DM and established cardiovascular disease (10).

In China there are three SGLT2 inhibitors that have been approved for use: dapagliflozin, empagliflozin and canagliflozin (11). SGLT2i are currently recommended in China as an additional therapy in patients who are unable to achieve glycaemic control with metformin monotherapy (11, 12). SGLT2i use in high-risk cardiovascular patients reduced Major Adverse Cardiovascular Events (MACE) including those with established heart failure, and recommendation of use is in conjunction with consultation of an endocrinologist (11, 13). Severely impaired renal function is a contraindication and dosage adjusting is needed with impaired renal function (11). Sotagliflozin is the first dual SGLT2/1 inhibitor being approved to be used in China for glycated haemoglobin (HbA1c) improvement in diabetic patients, which is associated with reduced body weight and blood pressure (14).

## SGLT2i Safety and Adverse Events

There are numerous reports of potential safety issues associated with the use of SGLT2i including the rare but serious adverse events of diabetic ketoacidosis and necrotising fasciitis of the perineum. The report regarding the possible heightened risk of lower limb amputation and fracture with SGLT2i is conflicting.

## Hypoglycaemia

SGLT2i used as a monotherapy are all associated with a low risk of developing hypoglycaemia, and hypoglycaemia risk is raised when utilised as an add-on therapy with sulfonylurea or insulin (8, 9). Nevertheless, clinical trials in China found that SGLT2i monotherapy was not observed with increased hypoglycaemia risk (11).

## Mycotic Infections of Perineum and Urinary Tract Infections (UTIs)

The most well-documented adverse event of SGLT2i in comparison with placebo groups is mycotic infections, particularly amongst the female population (15, 16). Interestingly, a metanalysis found that risk of mycotic genital infections is lowered when SGLT2i combine with DPP4 inhibitors opposed to being used as monotherapy or used as an adjunct to metformin (17). Necrotising fasciitis of the perineum, called Fournier's gangrene is a significant adverse event, and post-marketing cases review confirmed lower incidence of 55 cases reported to the US FDA between 2013 and 2019 with three cases of fatality related to the complication (18).

## Diabetic Ketoacidosis (DKA) and Euglycemic DKA

The widespread use of SGLT2i in recent years has been associated with a slightly increased risk of diabetic ketoacidosis. (19, 20) The rates of DKA in major trials were 0.1–0.5% over 4–8 years (19–21), in concert with a large Australian observational study showing an incidence of 0.1% over 26 months (22). In comparison to non-SGLT2i antihyperglycemic medications, there was no statistically significant increase of risk in SGLT2i use for type 2 diabetic patients (23). The US Food and Drug Administration (FDA) and European Medicines Association (EMA) respectively issued drug safety reports on DKA risk in SGLT2i patients with DKA-like symptoms at health services (3). In light of the insulin-independent inhibition on glycaemia, many DKA cases present with mildly elevated or even normal blood glucose levels, leading to delayed diagnosis and management with an emerging term “Euglycemic DKA” (22). The risk of euglycemic DKA is significantly raised in the clinical settings of being acutely unwell, fasting, perioperative states or having excess alcohol consumption (24). Furthermore, confirmation of euglycemic DKA will only be reliable by measuring capillary blood ketone or blood beta-hydroxybutyrate levels because accurate measurement of urine ketone may be impaired by altered urinary ketone excretion (25). Five anecdotal cases suggests there may be an increased risk of Euglycemic DKA in Type 2 diabetic patients who are infected with SARS-CoV-2 infection (26). In balance of all adverse events data, SGLT2i is clinically safe with negligible albeit serious side effects to monitor, especially euglycemic DKA in the presence of both elevated ketonuria and normal ketonuria. Patients should be advised to withhold SGLT2i when they are unwell, fasting or at least 3 days preoperatively.

## Amputation

Canagliflozin in clinical trial (CANVAS) increased the risk of toe and foot amputation (HR 1.97; 955 CI 1.41, 2.75) (27), as well as a decrease in bone mineral density in another study (28). The increased risk of amputation seems to be inconsistent with report only in the CANVAS trial but not in empagliflozin or dapagliflozin trials, nor in the subsequent Canagliflozin and Renal Events in Diabetes with Established Nephropathy Clinical Evaluation (CREDENCE) trial with canagliflozin (19, 29). The increased risk associated with Peripheral Arterial Disease (PAD) when using Canagliflozin can inform physicians to minimise the risk of amputation in this group of patients (29). To date, there was no definitive data to suggest a correlation between SGLT2i and amputation (30).

## Chronic Kidney Disease

The best practise of SGLT2i use for patients with kidney disease is debated (31). The use of SGLT2i in mild to moderate (eGFR 30-60 ml) diabetic kidney disease is recommended because of associated reduction in albuminuria and long-term preservation of eGFR (31), leading to reduced risk of AKI and cardiovascular events (31). Dapagliflozin use in patients with T2DM and stage-3A CKD was also reported to bring about a positive benefit/risk relationship (32). There is little to no data to suggest SGLT2i use in severe/end stage renal failure (31). SGLT2i may lead to volume loss through its diuretic effects and may consequently cause hypotension (33), with some clinical trials demonstrating exaggerated volume depletion (11, 19, 34), while others demonstrating euvolumia (20, 21). A recent meta-analysis suggested that all SGLT2 inhibitors potentially prevent the development of AKI, despite adverse events related to hypovolaemia (35). The mechanism of AKI prevention is thought to be related to reduced renal hyperfiltration although an initial and transient decline in eGFR is usually followed by SGLT2i commencement (35). In summary, SGLT2i is clinically safe to use in diabetic patients with mild to moderate renal failure individuals.

## Cancer

SGLT-2i did not seem to increase the overall risk of cancer in patients with T2DM. In terms of global use of common SGLT-2 inhibitors including canagliflozin, dapagliflozin and empagliflozin, most research showed that there were no direct links between SGLT2i use and overall cancer risk. Two reviews particularly reported for canagliflozin and empagliflozin, demonstrating contrasted trend of cancer risk with a statistically significant decreased risk of gastric cancer but increased bladder cancer risk (36, 37). Detection bias (38) and the observed imbalance between empagliflozin and control in lower incidents of events (37) are accounting for the statistically significant associations seen with empagliflozin use increasing the incidence of bladder cancer. Detection bias are related to increasing bladder cancer risk arising from SGLT2i-induced recurrent genital tract infections secondary to their mechanism of action (39–41). The observed discrepancy between empagliflozin and control in lower incidence of events showed just an incidence of 48 cases of bladder cancer in 28,055 participants treated with SGLT2i, in comparison to an incidence of 58 cases of bladder cancer in 20,594 participants treated with placebo (37). In terms of decreasing gastric cancer risk, a recent meta-analysis also hinted a statistically significant decreased risk of gastrointestinal cancer in canagliflozin-treated patients (36). However, short follow-up times and low event rates in most clinical trials precluded the proper assessment of long-term gastrointestinal cancer risk in canagliflozin-treated participants. Furthermore, gastrointestinal cancer risk was not demonstrated in another meta-analysis (37). Long-term interventional studies are required to assess SGLT2i-associated cancer risk especially with bladder and gastrointestinal cancers.

### SGLT2 Inhibitors' Role of Primary Prevention Beyond Diabetes

Apart from well-documented benefit in diabetic patients as a monotherapy or add-on therapy, more recent evidence demonstrated that SGLT2i reduced major adverse cardiovascular events (MACE) and hospitalisation for heart failure (HHF) (42, 43). SGLT2i showed benefits on delaying the onset and worsening of renal complications by lowering serum creatinine, reducing albuminuria, and decreasing mortality associated with renal disease (44, 45). The cardiovascular and renal benefits seem to be unrelated to glycaemic control, making SGLT2i the most recommended class of medications for diabetic patients with significant cardiovascular or renal risks. These significant findings would postulate the potential primary preventive benefits of SGLT2 inhibitors in terms of cardiovascular outcomes. This postulation has been substantiated in a recent review with Metformin, which has been showed to have potential primary preventive role in reducing cardiovascular events in the healthy and non-diabetic elderly (46). In non-diabetes patients with heart failure, SGLT2i has shown positive impact on weight and blood pressure (38).

Taken together, evidence is mounting for reviewing the literature of SGLT2i use in both diabetic and non-diabetic population (Table 2), so as to substantiate our postulation of its potential primary preventive role in reducing events in healthy adults.

**Table 2:** Summary of SGLT2i preventive effects in DM and Non-DM patients.

	**DM**	**Non-DM**
CKD prevention	Empaglifozin ↓ eGFR, albuminuria. Empaglifozin vs. placebo ↓ risk of kidney disease, kidney deterioration, renal replacement therapy.	↓ The onset of end stage renal failure in patients with non-diabetic nephropathy/CKD by reducing the single nephron glomerular filtration rate (SNGFR). Cessation of SGLT2i will lead to SNGFR returning to its original GFR before SGLT2i use.
CVD prevention	Empagliflozin vs. placebo ↓ MACE by 14%, CVD mortality by 38%, all-cause mortality by 32%, HF hospitalisation by 35%. Canagliflozin vs. placebo ↓ MACE by 14%, + Dapagliflozin: non-significant reduction in MACE. → Significant effect of SGLT2i on MACE in DM patients with established CVD onlySGLT2i ↓ LV mass after 6 months, however no change in LVEF, LV end diastolic volume, LV end systolic volume.	Patients with NYHA class IIIV HF and an EF ≤ 40%: Dapagliflozin vs. placebo ↓ risk of a worsening HF and CVD mortality by 26%.
Hypertension prevention	SGLT2i vs. placebo ↓ 24-h, daytime and nocturnal BP (average systolic 5.5 mmHg & diastolic 1.5 mmHg)	SGLT2i might reduce both body weight and blood pressure in non-diabetic patients.
Dyslipidaemia prevention	SGLT2 vs. placebo ↑ HDLc, ↓ TG, ↑ LDLc	No studies in human yet
Obesity prevention	SGLT2 vs. placebo ↓ 270 calories daily, ↓ 3 kg after 24 weeks	No studies in human yet

## Prevention of CKD in DM and Non-DM Patients

Chronic Kidney Disease leads to many severe morbidity and mortality, with hypertension and diabetes being the commonest causes. Diabetic nephropathy is ranked the second in causing end-stage kidney disease in China, while the global incidence of diabetic microalbuminuria is 39% with the incidence of albuminuria being 3.1% annually (47). Thirty percentage of T1DM and 20% of T2DM in China eventually develop diabetic nephropathy and chronic kidney diseases (48), making it one of the most serious chronic complications and main causes of death in diabetic patients. In comparison with placebo, Empaglifozin showed a significantly decreased incidence of kidney disease, risk of CKD worsening, and risk of renal dialysis in diabetic patients (49). The relative risk of doubling urinary albumin/creatinine ratio was also significantly reduced by SGLT2i. Thus, ADA guidelines 2019 recommend the use of SGLT2i to decrease the risk of kidney disease progression and cardiovascular events in patients with T2DM complicated with CKD (50). The Chinese Clinical Guide 2019 for the Prevention and Treatment of Diabetic Renal Disease clearly stated that SGLT2i have reno-protective effects in addition to hypoglycaemic effects, especially when patients with diabetic nephropathy are unable to adequately reduce hyperglycaemia with metformin (51).

The mechanism of the reno-protective benefit of SGLT2i for diabetic patients has been proposed in a few recent studies (52–54) including improving glomerular hyperfiltration, reducing renal oxygen consumption and renal inflammatory reaction, restoring the mode of cellular energy metabolism. A recent review (55) discussed the indirect mechanism of reno-protective benefit with SGLT2i, which includes reducing hyperglycaemia, lowering blood pressure, decreasing uric acid, promoting weight loss, increasing glucagon level, reducing insulin level and promoting diuresis (55).

In non-diabetic population, SGLT2i has been found to delay the onset of end stage renal failure in patients with non-diabetic nephropathy (50) *via* the mechanism of reducing the single nephron glomerular filtration rate (SNGFR) and thus preventing hyper-filtration. Of important note is that cessation of SGLT2 inhibitors will lead to SNGFR returning to its original level prior to commencing the SGLT2 inhibitors (50).

SGLT2 inhibitors have proved to be reno-protective in non-diabetic CKD patients and have proved to delay the progression of kidney disease, lower the risk of cardiovascular events and improve survival rates, highlighting their use beyond the scope of only diabetic patients (55). The initial plasma volume reduction as well as the renal hemodynamic changes with SGLT2i therapy are observed with a small decrease in estimated glomerular filtration rate (eGFR) by 4–5 mL/min/1.73 m2 at the beginning followed by moderately increased or stabilised eGFR. SGLT2i has been demonstrated in slowing the worsening of renal function in the EMPA-REG OUTCOME trial where the eGFR decreased steadily over longer period in the placebo arm and stabilised in the empagliflozin arm (56). Consequently, empagliflozin prevented the decreased eGFR that occurs typically in diabetic patients. A meta-analysis of 48 RCTs, including 58,165 patients, has showed that SGLT2i statistically reduce albuminuria in comparison with placebo or active comparators. In a meta-regression analysis, the extent of reduction on albuminuria by SGLT2i was prone to be greater with relatively higher levels of albuminuria at baseline (57).

In summary, SGLT2i's reno-protective benefit raised the question whether there is a preventive role of SGLT2i in reducing chronic kidney diseases in healthy individual. There has been evidence that SGLT2i caused a transient decline in renal function but return to normal within a few weeks of commencing SGLT2i (57, 58). This piece of evidence provide assurance for carefully designed clinical trials to answer the question.

## Prevention of Cardiovascular Events in Both Diabetic and Non-Diabetic Patients With Heart Failure

Cardiovascular events are one of the most common and serious complications in both diabetic (59) and non-diabetic population reported by World Health Organisation (WHO). Recent United Kingdom studies have established that the mortality rate of cardiovascular events in diabetic patients is three times higher than that in non-diabetic patients (60). Chinese data has echoed the UK findings that 30% of diabetic patients have cardiovascular events, (61) with these events accounting for 20% of diabetes mortality in China (62). Cardiovascular events prevention has become a primary research focus in both diabetic and non-diabetic populations, with SGLT2i showing cardio-protective effect and reducing CV risks.

Three SGLT2i including empagliflozin, canagliflozin, and dapagliflozin have been evaluated in Cardiovascular Outcome Trials (CVOTs) 0.1 (9, 63–65). The EMPA-REG OUTCOME trial recruited 7,020 patients with T2DM and established CVD for 192-weeks follow-ups. The trial showed that the number of major adverse cardiovascular events (MACE) are significantly less frequent in patients receiving empagliflozin vs. those receiving placebo (HR 0.86, 95% CI 0.74–0.99; *p* = 0.04). Cardiovascular-related death, nonfatal cerebral infarction, and nonfatal myocardial infarction reduced by 14%, while the number of cardiovascular-related deaths reduced by 38%, as well as the number of hospitalised patients with heart failure reduced by 35%, and its mortality rate reduced by 32% in the SGLT2i group in comparison to the placebo group. Furthermore, the follow-up study demonstrated the ongoing significant cardiovascular benefit of empaglifozin from 30 to 90 days of treatment, as well as with the extension of treatment time compared with the placebo group. In the Canagliflozin Cardiovascular Assessment Study (CANVAS) trial, 10,142 patients with T2DM and high cardiovascular risk with 34.4% without prior CVD were randomly assigned to receive canagliflozin or placebo, with MACE being reduced by 14% (*p* = 0.02), but no significant reduction in individual components of the composite outcome (64). A significant group of low-risk patients including 59.4% of the 17,160 patients without established CVD were recruited in the Dapagliflozin Effect on Cardiovascular Events–Thrombolysis in Myocardial Infarction 58 (DECLARE-TIMI 58) trial. Dapagliflozin failed to demonstrate significant benefit in reducing MACE (65). Another study (1) compared the subgroup of DECLARE patients with known atherosclerotic CVD with the other SGLT2i CVOTs, and revealed a non-significant interaction and absent heterogeneity between the three CVOTs. A reduction in MACE by SGLT2i was observed only in patients with established CVD instead of low-risk patients (1).

A subgroup analysis of Canagliflozin and Renal Events in Diabetes with Established Nephropathy Clinical Evaluation (CREDENCE) trial in patients without CVD (*n* = 2,181, 49.6%), showed that canagliflozin significantly decreased the risk of MACE by 20% (*p* = 0.01) with consistent reduction in both primary and secondary prevention group (HR 0.68, 95% CI 0.61–0.94 and HR 0.85, 95% CI 0.69–1.06, respectively; p interaction 0.25) (66). Another CVOTs finding regarding the rates of hospitalisation for heart failure (HHF), which was significantly reduced by 30–35% with SGLT2i (19, 51–53). The onset of the SGLT2i benefit was apparent within the first weeks after randomisation, indicating a hemodynamic mechanism rather than anti-atherosclerotic effects. Dapagliflozin and Prevention of Adverse Outcomes in Heart Failure trial (DAPA-HF) recruited 4,744 patients with New York Heart Association class II-IV HF and an ejection fraction of 40% or less received dapagliflozin or placebo, with the primary endpoint being a composite of worsening HF or cardiovascular death. Dapagliflozin significantly reduced risks of worsening heart failure and cardiac death compared with placebo irrespective of the patients' diabetic status (HR 0.74, 95% CI 0.65–0.85; *p* < 0.001) (34) The mechanism of SGLT2i on human myocardial function is poorly understood, and most of the observational studies and clinical trials seem to suggest that SGLT2i can improve left ventricular (LV) diastolic function in diabetic patients (64, 65, 67). A recent RCT of 97 patients with T2DM and coronary artery disease defined the primary endpoint as the 6-month change in LV mass measured by cardiac MRI. Empagliflozin was associated with a significant reduction in LV mass without affecting LV ejection fraction, LV end-diastolic volume, LV end-systolic volume (67). There are ongoing RCTs investigating the effects of SGLT2i on LV remodelling in patients with T2DM and HF (68, 69). Another SGLT2i RCT used impedance cardiography to demonstrate that 12 weeks of dapagliflozin had no significant effects on cardio-dynamic parameters related to blood flow, systolic function, circulatory function, and fluid status in comparison to placebo (69).

Taken together, SGLT2i has strong evidence of prevention of MACE and HHF in diabetic patients, whereas the evidence is not consistent with the low-risk non-diabetic patients. It is worthy to proceed with a RCT to examine the preventive role of SGLT2i in non-diabetic low-risk population, especially the prevention of MACE and cardiac death as being observed in DAPA-HF trial (34).

## Prevention of Hypertension in Healthy Individuals

Recent review on SGLT2i BP lowering effects in diabetic patients with hypertension (70) has demonstrated consistent and clinically significant reductions in 24-h, daytime and nocturnal BP. SGLT2i appears to reduce morning, evening and nocturnal home BP in patient subgroups with higher BMI and baseline BP. The mechanism of this BP lowering effects is thought to be *via* the inhibition of SGLT2i on sodium reabsorption, resulting in natriuresis, osmotic diuresis and subsequent BP reduction by plasma and extracellular volume contraction (70). BP reduction was observed in Phase III studies with the average 5.5 mmHg decrease in systolic and the average 1.5 mmHg decrease in diastolic BP (71). In similar studies (33, 72), SGLT2i demonstrate an average reduction of systolic BP by 2.46 mmHg and diastolic BP by 1.46 mmHg, and average reduction of 24-h ambulatory systolic and diastolic BP by 3.76 mmHg and 1.83 mmHg, respectively. These results were consistent across different SGLT2i in comparison with placebo or with other hypoglycaemic-medications in patients already receiving antihypertensive therapy (71, 73). BP reduction appears independent from improved glycaemic control in all these studies. In studies of diabetic patients with CKD, SGLT2i was still effectively lowering BP even at negligible dosage (74), and BP was reduced significantly without sympathetic nervous system activation by not increasing heart rate (74), but reduced preload and afterload leading to a reduction of cardiac workload, myocardial oxygen demand and an improved LV function. Nevertheless, BP reduction is unlikely to explain all cardiovascular benefit. As demonstrated in recent review (70), many clinical trials have documented BP reductions in SGLT2i-treated patients with T2DM and hypertension (HTN), being independence of glycaemic control (74). Furthermore, recent evidence suggested that SGLT2i might reduce both body weight and BP in non-diabetic patients (75, 76). One would postulate whether these BP-reduction benefit will extend to the healthy and relatively low risk population as a potential preventive treatment modality. A clinical trial in this population group will be feasible if the safety of SGLT2i use in this population group can be assured to be minimal, as described in the section of SGLT2i use and safety.

## Contribution or Prevention of Dyslipidaemia in Healthy Individuals Treated With SGLT2 Inhibitors

There were inconsistent results in numerous clinical trials with diabetic patients regarding the effects of SGLT2i on dyslipidaemia. One study showed that SGLT2i raised HDL cholesterol and reduced triglycerides levels (77), however these benefits were associated with raised LDL cholesterol levels (21). It remained unclear whether the increased HDL cholesterol level was associated with improved reverse cholesterol transport. The dapagliflozin RCT trial was unable to significantly demonstrate the benefit of raised HDL cholesterol level in association with improved reverse cholesterol transport (78). In an animal study with diabetic mice, canagliflozin increased circulating LDL cholesterol and reduced triglycerides, as well as increased lipoprotein lipase activity, decreased postprandial lipemia, accelerated clearance of radiolabelled VLDL, and delayed turnover of labelled LDL from circulation (79). Another study pointed out that SGLT2 inhibition with empagliflozin reduces liver triglycerides and total cholesterol levels in prediabetic ob/ob-/- mice (80). Overall, SGLT2i impact on dyslipidaemia in both human and animal studies is inconclusive to be of benefit and will be therefore unlikely to contribute significantly to the prevention of cardiovascular events.

## Prevention of Obesity/Causing Weight Loss in Healthy Individuals

Obesity is well established to be an independent risk factor for cardiovascular disease (81). SGLT2i promote weight loss *via* glycosuria and negative energy balance, thereby leading to significant weight loss (81). SGLT2i use is associated with reduction of MACE through clinically significant weight loss. By excreting average 70 g of urinary glucose daily, SGLT2i lead to a significant loss of average 270 calories daily, consequently significant weight loss of average 3 kg over 3 months (71). This reduction is consistent across all RCTs and CVOTs with SGLT2i being used in monotherapy or in combination with other glucose lowering medication (GLM) (20, 21, 27, 82). Analysis of body composition by dual-energy x-ray absorptiometry showed that average 65% of the total weight loss by dapagliflozin was fat mass (83). However, another study using different methods of body composition analysis showed inconsistent results (84). In a MRI sub-study, dapagliflozin reduced both visceral and subcutaneous adipose tissue (83). In another SGLT2i study (79), weight loss becomes stagnant and appears much less than expected calorie loss from the amount of daily glycosuria without changing energy intake. The weight loss by SGLT2i is stabilised at 24 weeks with quantitatively similar weight loss compared to GLP-1RA (79), indicating the mechanism of weight loss by SGLT2i with a normal/increased caloric intake. In another study, the SGLT2i-induced weight loss in the first week of treatment seems to persist for a longer period of time (21). There is evidence that SGLT2i cause glycosuria and negative energy balance with consequent weight loss (85). Most of these studies demonstrated the weight loss benefit by SGLT2i in diabetic and high-risk patients, and it should be important to explore the potential weight loss benefit in non-diabetic, low-risk and even healthy patients with a RCT.

## SGLT2i Role of Primary Prevention in Conclusion

In summary, SGLT2 inhibitors reduce MACE in patients with known atherosclerotic cardiovascular disease, cardiovascular risk factors including hypertension, dyslipidaemia, overweight/obesity, and have a profound effect on preventing hospitalisation for heart failure, as well as reducing the risk of worsening estimated glomerular filtration rate, end-stage kidney disease, or renal death. However, the majority of these evidences concerned diabetic patients or high CVS risk patients with only small numbers of low-risk/heathy individuals in studies of non-diabetic, coronary arterial diseases and heart failure patients. Taking all the evidences together, there is a need for consideration of SGLT2i as a potential agent for primary prevention clinical trial in low-risk of cardiovascular and renal diseases or even healthy population with research design focusing on ensuring its safety and minimising potential side effects. A carefully-designed protocol for this SGLT2i double-blind randomised controlled primary prevention trial should answer the ultimate goal of exploring its prevention role in reducing events and improving quality of life in the healthy individual.

## Author Contributions

DX, OC, CW, CH, JA, DY, XL, WC, YL, CR, and HX reviewed the final manuscript. DX, DY, XL, WC, YL, CR, and HX designed the manuscript. DX, OC, CW, and CH drafted the initial manuscript. All authors contributed to the article and approved the submitted version.

## Conflict of Interest

The authors declare that the research was conducted in the absence of any commercial or financial relationships that could be construed as a potential conflict of interest.

## Publisher's Note

All claims expressed in this article are solely those of the authors and do not necessarily represent those of their affiliated organizations, or those of the publisher, the editors and the reviewers. Any product that may be evaluated in this article, or claim that may be made by its manufacturer, is not guaranteed or endorsed by the publisher.
